# JAK2V617F: Is It Sufficient as a Single Player in Splanchnic Venous Thrombosis?

**DOI:** 10.1155/2015/373490

**Published:** 2015-03-24

**Authors:** Pratibha Dhiman, Priyanka Saxena

**Affiliations:** Department of Hematology, Institute of Liver and Biliary Sciences, New Delhi 110070, India

## Abstract

Splanchnic venous thrombosis (SVT) includes thrombosis of the hepatic, portal, and mesenteric venous system. Myeloproliferative neoplasms (MPNs) are important factors of SVT in adults. Addition of JAK2V617F mutation in WHO criteria for diagnosis of MPNs has made this test a useful tool for diagnosis. JAK2 is an intracytoplasmic tyrosine kinase that plays a critical role in signal transduction from multiple hematopoietic factor receptors. The mutation is found frequently in patients with SVT; many such patients have no other manifestations of an MPN. Although the correlation of JAK2V617F mutation with thrombotic risk in MPNs has been shown in many studies, the impact of presence of additional thrombophilic factors in these cases is yet not known. As the management of MPNs remains highly dependent on the patient's thrombotic risk, it is important to assess the thrombotic risk factors in detail. Here, we report two cases of JAK2V617F positive MPN who also had other thrombophilic conditions and presented with recurrent thrombosis.

## 1. Introduction

Abdominal venous thrombosis represents a manifestation of unusual thrombosis and although rare, these thrombotic events are often severe, accounting for important morbidity and mortality. It may be associated with a wide spectrum of underlying disorders, either local or systemic. Local factors comprise abdominal infections or inflammations, cancer, cirrhosis, abdominal trauma, and recent surgery [1]. The predisposing systemic conditions include diseases such as myeloproliferative neoplasms (MPNs), chronic inflammatory disorders, paroxysmal nocturnal hemoglobinuria (PNH), Behcet's disease, and also acquired thrombophilic conditions such as the antiphospholipid syndrome, pregnancy and puerperium, and the intake of oral contraceptives. The role of deficiencies of the natural anticoagulant proteins antithrombin, protein C, and protein S is less certain [2]. MPNs, whether overt or latent, represent a major risk factor for the development of thrombosis in the portal, mesenteric, and hepatic areas [3]. Until recently the diagnosis of occult MPN in patients presenting with hepatic or portal vein thrombosis was difficult to establish and was often overlooked. In recent years, the discovery of the JAK2V617F mutation and its more frequent routine use in the diagnosis of MPN is a major advance. The presence of JAK2V617F strongly correlates with the bone marrow histopathology of MPN and has a high predictive value for the detection of MPN [4]. The results of studies regarding correlation of JAK2V617F mutation with thrombosis are variable. A significant association of JAK2 mutation with thrombosis was evident in half of these studies whereas no such correlation was documented in the remaining studies, although the analysis of larger series had shown that JAK2V617F is associated with an increased risk of venous and arterial thrombosis and for thrombosis at presentation. But it is still unclear if this particular association of JAK2V617F with thrombosis is independent of other confounding factors with known significant effect [5] which emphasize the need to analyse other thrombophilic factors even in the presence of this mutation to strategize the treatment plan. We hereby report two cases of JAK2V617F positive MPN who presented with progression of thrombosis on treatment and were found to have an additional prothrombotic factor.

## 2. Case Presentation

### 2.1. Case 1

**Figure 1:**
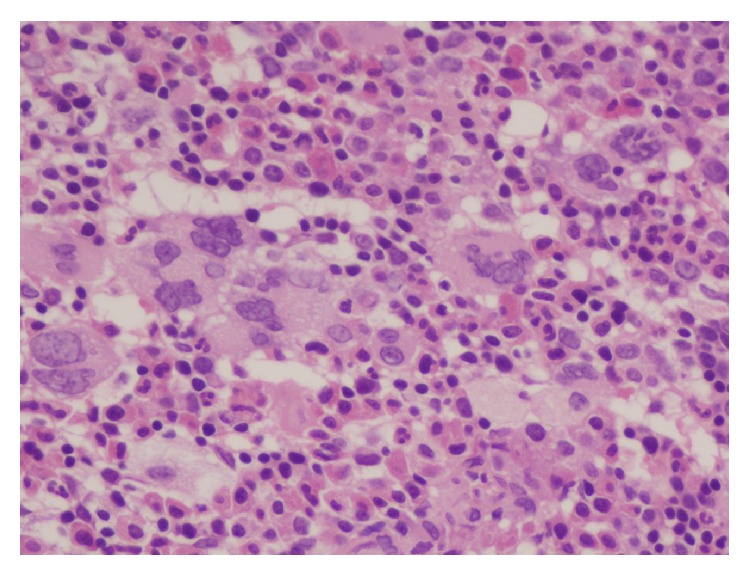
Photomicrograph of bone marrow biopsy showing hypercellular marrow with megakaryocytic hyperplasia and hyperlobulated forms (400x).

A 40-year-old female presented with dyspepsia, pain with discolouration of finger tips, pain in left leg, and abdominal pain for 20 days. There was no history of fever, jaundice, headache, bleeding from any site, and major surgery. On examination, liver was palpable 3 cm below the right costal margin and spleen was palpable 8 cm below the left costal margin with no significant lymphadenopathy. On investigations, complete hemogram showed Hb of 13 g/dL, WBC count of 10,900/*µ*L, platelet count of 522 × 10^9^/L, and differential count P 71, L20, M06, and E03. Contrast enhanced CT (CECT) of abdomen revealed thromboses of main and branches of portal vein with massive splenomegaly and normal liver architecture. USG Doppler of both lower limbs did not reveal any abnormality. In view of thrombocytosis with splanchnic thrombosis, sequencing for JAK2 was done which showed homozygous JAK2V617F mutation. Bone marrow examination showed moderately hypercellular marrow with megakaryocytic hyperplasia and predominance of hyperlobulated forms with clustering at places consistent with myeloproliferative neoplasm possibly ET (Figure 1). Bcr-Abl by reverse transcription polymerase chain reaction (RT PCR) was negative. Patient was started on anticoagulation with cytoreductive therapy in the form of hydroxyurea. Her abdominal pain decreased and follow-up counts showed significant reduction in platelet count and spleen size. Also INR (international normalised ratio) remained in the therapeutic range of 2-3. After 5 months, she presented with abdomen pain along with heaviness. On examination, spleen was increased to 6 cm below the left costal margin again. Her hemogram findings were Hb of 13.1 g/dL, WBC count of 4100/*µ*L, and platelet count of 160 × 10^9^. PT/INR on warfarin was 31.9/2.46. Her CECT of abdomen revealed extension of thrombosis up to superior mesenteric vein. As the thrombosis extended on treatment, we investigated additional prothrombotic factors after 6 weeks of the event and after stopping anticoagulation. On investigations, protein C level was 92.5%, antithrombin was 94.4%, and protein S was 33.6%. Factor V Leiden, methylenetetrahydrofolate reductase (MTHFR) polymorphism, and prothrombin G20210A were not detected. Workup for lupus anticoagulant was negative. To rule out an acquired cause for protein S deficiency, family screening was done which showed her sister to be protein S deficient but she was asymptomatic. In this case, protein S was the hereditary thrombophilic factor which has amplified the risk of thrombosis.

### 2.2. Case 2

A 46-year-old female presented with pain and distension of abdomen for 1 month. She was a known diabetic on treatment. There was no history of fever, headache, jaundice, and bleeding from any site. On examination, mild pallor with spleen was palpable 2 cm below the left costal margin. Her complete hemogram revealed Hb of 10.7 g/dL, WBC count of 8800/*µ*L, and platelet count of 500 × 10^9^/L. Her anemia was attributed to coexisting iron deficiency. CECT of abdomen showed thrombus in main, right, and left branches of portal vein with chronic thrombus in splenic and superior mesenteric vein with splenomegaly and grade 1 fatty liver. On further investigations, she was found to be heterozygous for JAK2V617F mutation. Her bone marrow examination showed hypercellular marrow with hyperlobulated megakaryocytes suggestive of MPN possibly ET. Bcr-Abl by RT PCR was negative. She was started on hydroxyurea and anticoagulation. Her INR was stable at 2.26–2.48. After 2 months she came with joint pain, swelling of left side of neck for 2 days which was painful and progressively increasing, and breathlessness. On examination, she had tachycardia and bilateral dullness on chest percussion which on on X ray of the chest was confirmed to be bilateral pleural effusion. Her USG of neck showed complete thrombus of left subclavian vein and partially thrombosed axillary vein. CT pulmonary angiography revealed thromboses of bilateral lower lobe segmental branches of pulmonary artery (right more than left) with a small peripheral wedge shaped infarct seen in right lower lobe. *D* dimer was 1 *µ*g/mL (positive). She was started on low molecular weight heparin. In this case, also progression of thrombosis to distant sites despite being on adequate anticoagulation was not explainable. Therefore, we did thrombophilic workup after 6 weeks of the event and off anticoagulation, which showed protein C 82%, protein S 75%, and antithrombin 113%. Factor V Leiden, prothrombin G20210A, MTHFR polymorphism, and workup paroxysmal nocturnal hemoglobinuria were negative. Antiphospholipid IgM, anticardiolipin IgM antibodies, and confirmatory test for lupus anticoagulant (LA) were positive. ANA was also positive. Rheumatology opinion was taken and as per the disease activity, patient was planned to start on hydroxychloroquine.

## 3. Discussion

The aim of reporting these two cases is to stimulate the large group study for defining the proper role of screening for additional thrombophilic factors in patients of JAK2 positive MPNs. Both patients were <60 years of age, one case was heterozygous, and the other was homozygous for JAK2V617F mutation. Both the cases were started on cytoreductive therapy and anticoagulation owing to high risk MPN. Despite the decrease in platelet count and spleen size with therapeutic INR, these patients had progression of thrombosis which prompted us to work up for other prothrombotic conditions which can exacerbate the thrombotic risk of JAK2V617F positive MPN.

JAK2V617F mutation is identified in most patients with polycythemia vera (PV) (95%) and in a significant proportion of patients with ET (50–60%) and primary myelofibrosis (50–60%) [6]. This mutation has been found to occur in 34–36% of SVT patients, 33–40% of hepatic vein thrombosis (HVT) patients, and 17–41% of portal vein thrombosis (PVT) patients [7, 8]. In an Indian study, presence of JAK2V617F mutation was noted in 19% of patients with SVT (18% with PVT and 20% with HVT) [3]. There are contradictory reports about the association between JAK2 mutation and thrombosis in patients with myeloproliferative neoplasm. An association between JAK2V617F mutation and risk of venous thromboses has been described in a large study that compared 362 mutant with 414 wild type (WT) patients with ET, although no information was provided about homozygous patients [9]. In addition, another study of 179 ET patients found that the frequency of thrombosis in JAK2V617F mutation was significantly increased and was comparable to that of PV patients [10].

Also in a study by Vannucchi et al. the frequency of thrombosis at diagnosis was double in heterozygous ET patients compared to that in WT patients (21.7% versus 10.5%), although the difference did not attain the significance level; however, the difference reached the significance level if all mutant (heterozygous plus homozygous) ET patients were compared with a WT counterpart [11]. Wolanskyj et al., on the other hand, observed 150 patients with ET in which 73 were V617F+, and the incidence of thrombotic complications was not different from their V617F− counterparts [12]. The influence of gender on JAK2V617F allele burden has been studied by Stein et al. who showed that, despite their younger age, less prevalent dyslipidemia or smoking history, lower white blood counts, and lower JAK2V617F allele burden, women had higher rates of abdominal venous thrombosis and comparable rates of all vascular complications [13]. The correlation of thrombosis with other mutations associated with MPNs is also studied. Rotunno et al. have studied 576 patients with ET and concluded that patients carrying the CALR mutation had a lower risk of thrombosis than JAK2- and MPL-mutated patients [14]. Also the incidence of CALR mutations in patients with splanchnic vein thrombosis was reported by Haslam and Langabeer, who studied 144 patients of SVT for JAK2V617F and CALR exon 9 mutations. The JAK2V617F was detected in 18.8% (27/144) of patients whereas none of the patients was positive for CALR exon 9 [15].

The impact of JAK2V617F on thrombotic risk likely extends beyond its effect on platelets. The presence of JAK2V617F mutant liver endothelial cells but not hematopoietic cells in patients with Budd-Chiari syndrome suggests that expression of JAK2V617F also directly affects nonhematopoietic cells to augment thrombotic risk [16]. Literature regarding the presence of other prothrombotic factors is scarce. Sokolowska et al. examined 32 patients with JAK2V617F positive ET and looked for the coexistence of other thrombophilic markers. They studied mutations including factor V Leiden, prothrombin, and MTHFR and also evaluated plasma levels of fibrinogen, factors VIII and XII, antithrombin, protein C, protein S, and serum level of homocysteine. Among these patients, protein S deficiency was diagnosed only in one person who, besides erythromelalgia, presented with one episode of transient ischemic attack. Two patients were diagnosed to have factor V Leiden mutation and in one ET patient, who had thrombotic complications, a homozygous MTHFR C677T allele was identified [17].

One of our cases had protein S deficiency as an additional prothrombotic factor. A decrease in the free protein S is reported in patients with ET due to protein S cleavage by a protease from platelets [18] and it returned to normal values in ET subjects receiving hydroxyurea treatment and with a normal platelet count [19]. In our patient, as family screening was positive and despite the normal platelet count, patient had progression of thrombosis, and protein S has certainly increased the thrombotic risk of JAK2V617F positive MPN itself.

The second case was detected to have ET with antiphospholipid syndrome. This patient also presented with progression of thrombosis despite therapeutic INR which actually can be an overestimation of INR due to LA. Valle et al. have shown that INR determinations obtained with a recombinant PT reagent substantially overestimate the actual degree of anticoagulation of most LA patients and that this is due (irrespective of correct international sensitivity index (ISI) assignment) to interference of lupus anticoagulant IgG in PT assays [20].

We suggest that one should look beyond JAK2 in patients of MPNs who present with thrombosis of unusual vascular territory because multiple factors presented together can accelerate thrombus formation and also treatment modalities will differ in case of additional prothrombotic factors.
